# Incidence of Scrub Typhus in Rural South India

**DOI:** 10.1056/NEJMoa2408645

**Published:** 2025-03-13

**Authors:** Carol Devamani, Neal Alexander, Daniel Chandramohan, John Stenos, Mary Cameron, Kundavaram PP Abhilash, Punam Mangtani, Stuart Blacksell, Huong Thi Thu Vu, Winsley Rose, Wolf-Peter Schmidt

**Affiliations:** 1 Department of Child Health 3, Christian Medical College, Vellore, India; 2 MRC International Statistics and Epidemiology Group, London School of Hygiene and Tropical Medicine, London, UK; 3 Department of Disease Control, London School of Hygiene and Tropical Medicine, London, UK; 4 Australian Rickettsial Reference Laboratory, Barwon Health, Geelong, Australia; 5 Department of Emergency Medicine, Christian Medical College, Vellore, India; 6 Department of Infectious Disease Epidemiology, London School of Hygiene and Tropical Medicine, London, UK; 7 Mahidol-Oxford Tropical Research Medicine Unit (MORU), Faculty of Tropical Medicine, Mahidol University, Bangkok, Thailand; 8 Centre for Tropical Medicine and Global Health, Nuffield Department of Medicine, University of Oxford, Oxford, United Kingdom; 9 National Institute for Control of Vaccines and Biologicals, Ha Noi, Vietnam

## Abstract

**Background:**

Hospital studies suggest that scrub typhus is a leading cause of severe undifferentiated fever in endemic regions across Asia, but the population-based incidence of infection and illness has not been studied.

**Methods:**

We systematically assessed 32,279 individuals from 7,619 households living in 37 villages, in a high scrub typhus endemic area, Tamil Nadu, India. Participants were visited every 6-8 weeks over two years for acute febrile illness. A venous blood sample was taken in those reporting a fever since the last visit. A sub-cohort of 5903 participants underwent annual blood sampling.

**Results:**

During 54,588 person-years, we observed 6175 fever episodes, with a blood sample taken in 4474 (72%). Of these, 328 (7.3%) met the case definition (ELISA or PCR positive). The incidence of scrub typhus was 6.0/1000 person-years (95% CI 4.8, 7.5). Seventy-one cases (21.6%) were hospitalized (incidence rate 1.3/1000 person-years, 95% CI 1.0, 1.7). Twenty-nine cases (8.8%) were severe (respiratory failure, encephalitis, kidney failure and/or shock), an incidence of 0.5/1000 person-years (95% CI 0.3, 0.8). In the sub-cohort, the incidence of seroconversion, independent of any symptoms, was 81.2/1000 person-years (95% CI 70.8, 91.6). Older age groups and women showed a higher incidence, but the risk of severe infection was higher in men. ELISA-IgG seroprevalence in 5602 participants tested at baseline was 42.8% (95% CI 35.8%, 50.2%). Baseline IgG sero-positivity did not protect against clinical illness but was associated with reduced disease severity.

**Conclusion:**

We describe the burden of scrub typhus in this endemic area including incident asymptomatic infections.

(Funded by UK Research and Innovation, Medical Research Council. Trial Reg: NCT04506944 (clinicaltrials.gov)).

Scrub typhus is an acute febrile infection, endemic in large parts of South Asia, East Asia and Southeast Asia,^1^ with cases also reported from the Middle East,^2^ Chile^3^ and East Africa.^4^ Severe illness is characterised by acute respiratory distress syndrome (ARDS), shock, renal failure and meningo-encephalitis.^5^ Scrub typhus is caused by intracellular bacteria of the genus *Orientia*, with *O. tsutsugamushi* being the dominant species in Asia.^6^ Strain diversity of *O. tsutsugamushi* limits immunity against re-infection.^7^ Orientiae are transmitted by the bite of trombiculid mite larvae (chiggers), leaving a characteristic eschar at the inoculation site.^8^ Hospital-based studies indicate that scrub typhus is a common cause of severe undifferentiated fever throughout Asia.^5,9–12^ Although doxycycline and azithromycin, alone or in combination,^13^ are effective against *O. tsutsugamushi*, lack of awareness often leads to failure to initiate treatment.^14^ The population-based incidence of infection and illness has not been systematically studied, hampering efforts to estimate the burden of infection.^12,15^ A Malaysian study from 1976 reported an annual incidence of clinical infection of 12 per 1000 among plantation workers.^16^ Population-based studies found incidence rates of serological infection ranging from 5 to 20 per 1000 person-years.^17–19^ However, the proportion of infections leading to clinical illness is unclear, limiting the applicability of serological data to calculate disease burden. There is also a lack of data on age and sex distribution by illness severity, and on the immunity of infection. We enrolled a population-based cohort in an endemic setting in South India to estimate these epidemiological parameters.

## Methods

### Enrolment and follow-up of participants

This was a population-based cohort study over two years in 37 rural villages of two districts (Vellore and Ranipet) in Tamil Nadu, India, where scrub typhus mainly occurs from August to February.^20^ Villages were eligible for enrolment if: 1) earlier studies had indicated a sero-prevalence of *O. tsutsugamushi* infection of at least 15% (n= 28 villages),^10,21^ or 2) at least two scrub typhus cases from a village were admitted to the study institution (Christian Medical College, Vellore) between 2016 to 2019 (n= 9). All residents who expected to reside for at least 6 months in selected villages were requested to take part in the study. To estimate the incidence of serological infection, one participant aged 10+ years per household present at the time of enrolment was randomly selected and asked to take part in a sub-cohort (sero-cohort). Enrolment was conducted from February to July 2020. As an open cohort, previously unenrolled households or new members within enrolled households were eligible for enrolment throughout follow-up.

Written consent for enrolment was obtained at the household level from the household head. Individual consent/assent was obtained before taking a blood sample. Households were visited every 6 to 8 weeks for two years, from August 2020 to July 2022. At each visit, participants were asked whether they had fever since the last visit or in the previous 2 months, whichever was shorter. For those not present, other household members were asked about their fever history. If a whole household was not present, an attempt was made to contact the household by phone. A venous blood sample was taken from all those reporting a fever, with administration of a brief questionnaire on clinical details including self-reported comorbidities. Excluded from blood sampling were PCR positive SARS-CoV-2 cases and cases with an identifiable cause of fever (e.g., cellulitis). Whenever appropriate, a study nurse examined cases for eschars. If feasible, a venous blood sample was collected from an asymptomatic control within the case or a neighboring household. Acute fever cases were visited by a study nurse on the same day and, if indicated, advised to seek treatment from a health care provider of their choice. For ongoing fevers, we aimed to take a second blood sample 4-6 weeks later (convalescent sample).

Participants enrolled into the sero-cohort underwent blood sampling at enrolment and between March and June of each year. If any participant died during the study, a study nurse administered an adapted verbal autopsy^22^ to household members to establish whether the death was associated with a febrile illness.

The study was approved by Christian Medical College’s Institutional Review Board (Ref: 11726) and London School of Hygiene and Tropical Medicine’s Research Ethics Committee (Ref: 16573). The study is registered at clinicaltrials.gov (NCT04506944), the protocol and statistical analysis plan can be found at nejm.org.

The study coincided with the first three SARS-CoV-2 waves in India. Consequently, the originally intended sample size of 40,000 had to be lowered to about 32,000 due to movement restrictions in February/March 2020. For the same reason, one follow-up round was cancelled in May/June 2021.

### Laboratory testing and case definitions

All blood samples from febrile cases (acute or convalescent) were tested for *O. tsutsugamushi* IgM and IgG using ELISA. All samples taken during the fever (“acute samples”) were tested for *O. tsutsugamushi* using PCR. All samples from the sero-cohort were tested for IgG using ELISA. As *Orientia*-specific antibodies can be present at baseline due to past infections, indirect immunofluorescence assays (IFA) were performed to confirm a new infection in paired samples where IgG ELISA was positive at both time points.^18,23^ Reversion from IgG ELISA positive to negative was treated as absence of infection without IFA confirmation. In the sero-cohort, IFA was restricted to a random sample of 25% of IgG ELISA-positive sample-pairs. The Supplement contains more details on laboratory methods.

The clinical case definition was: self/carer-reported febrile illness plus 1) a positive scrub typhus IgM ELISA, or 2) a positive qPCR for *O. tsutsugamushi*, or 3) a typical eschar if no blood sample could be obtained. A case with a positive IgM ELISA or an eschar if no blood test could be done was defined as “probable”. A case with positive PCR, or a case with a positive IgM ELISA and either an eschar, or a four-fold or greater increase in IFA or IgG ELISA seroconversion (from acute to convalescent sample) was defined as “confirmed”. Severe scrub typhus was defined as the presence of significant organ dysfunction (Table S2), in particular lung (SO_2_< 92%), CNS (focal neurological deficit, elevated white blood cell counts in cerebrospinal fluid, or seizure), cardiovascular (shock, myocarditis) and kidneys (creatinine ≥ 3.0), and included adverse pregnancy outcomes (miscarriage). For the sero-cohort, serological infection was defined independent of symptoms as: 1) IgG ELISA seroconversion, or 2) a four-fold or greater increase in IFA titer to at least 1:128.

### Statistical analysis

The incidence rate of clinical infection was calculated as the number of scrub typhus cases per 1000 person-years. For an individual, time at risk started 2 months before the first follow-up visit and ended with the last visit date at which the individual was still in the study, excluding intervals during which the individual was not observed. After scrub typhus infection, an individual was considered not at risk for 6 months, reflecting the short-lived protection against re-infection.^24–27^ Estimates were adjusted for within-village correlation using jackknife,^28^ which implicitly accounts for lower level clustering of episodes at household and individual level.^29^ Logistic regression was used to estimate the probability of IgM-positivity in fever cases where a blood sample was not available (see Supplement). In addition, some people without fever can be IgM positive due to previous asymptomatic infections.^18^ The clinical incidence rate was therefore adjusted for the prevalence IgM positivity in asymptomatic controls using population attributable fractions (see Supplement).

Risk factors were evaluated using Poisson regression (age- and sex-adjusted). Due to concerns regarding the willingness to report fevers and provide blood samples in the context of SARS-CoV-2 control measures, we conducted an unplanned subgroup analysis where the estimation of the incidence of clinical infection was restricted to participants of the sero-cohort.

The incidence of serological infection independent of any clinical symptoms in the sero-cohort was calculated separately for subgroups who were baseline negative (IgG ELISA seroconversion) or baseline positive (≥four-fold IFA titer increase). Sero-incidence was calculated using complementary log-log models with the time between blood samples as denominator and robust standard errors to account for within-village correlation.^30^ Individuals without midline samples were excluded. To account for demographic differences between the sero-cohort and the main cohort (Figure S1), sero-incidence was age/sex/year standardised using the main cohort demographics (see Supplement).

## Results

### Serological sub-cohort

**Figure 1:**
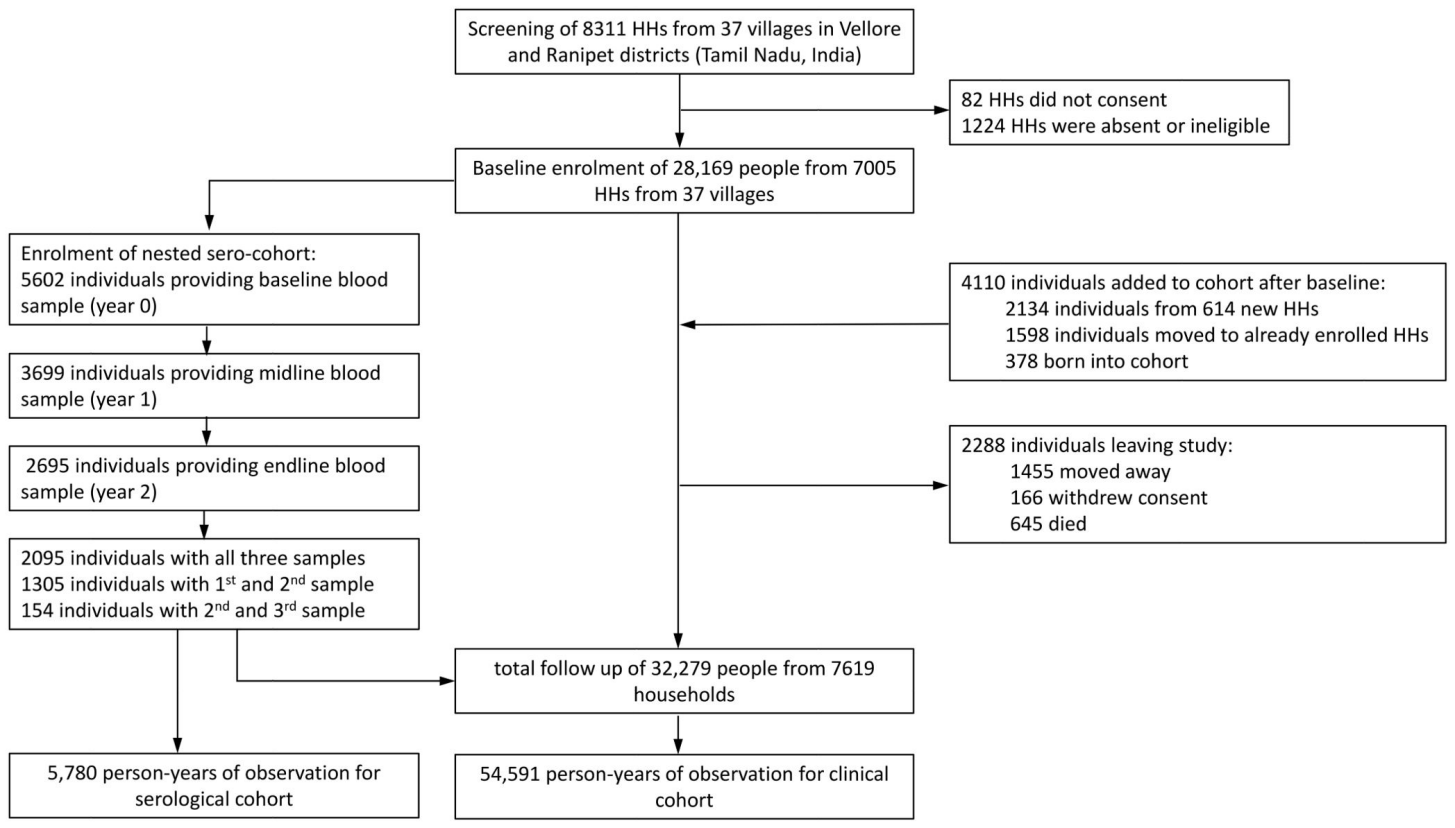
Study flow diagram

**Figure 2:**
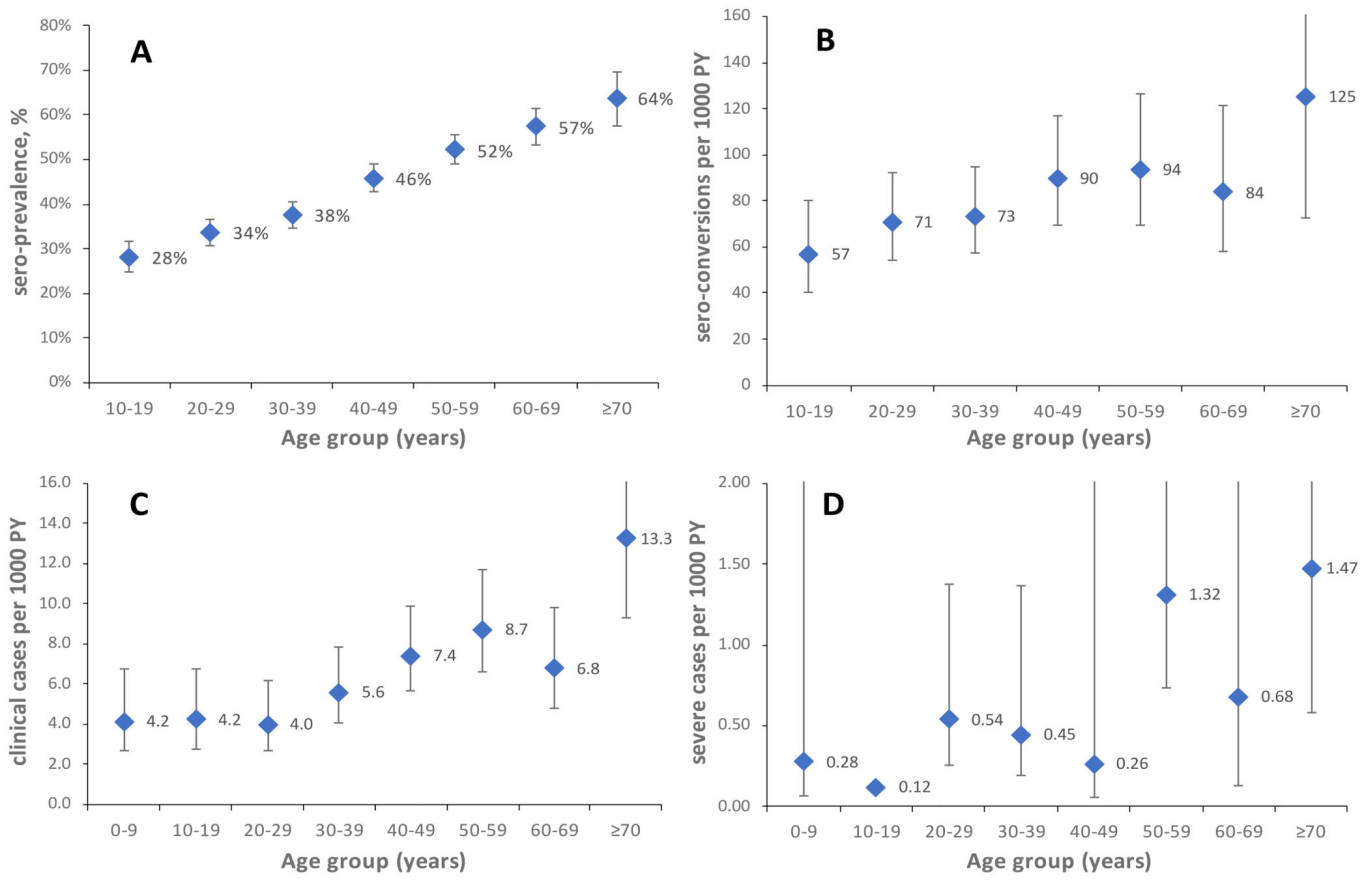
Scrub typhus sero-prevalence (A), sero-incidence (B), incidence of clinical infection (C) and incidence of severe infection (D) by age.

Overall, 5903 participants of the sero-cohort provided at least one sample. We collected a baseline sample in 5602 participants (Figure 1), showing an ELISA-IgG seroprevalence of 42.8% (95% CI 35.8%, 50.2%). Seroprevalence increased with age (Figure 2A). 3554 participants provided at least 2 consecutive samples. We observed 265 seroconversions in 3238 initially sero-negatives and an age/sex/year-standardised incidence of 81.2/1000 person-years (95% CI 70.8, 91.6). The incidence of seroconversion increased with age (Figure 2B) and was higher in females than in males (age-adjusted RR 1.5, 95% CI 1.2, 1.9). Incidence was 1.6 times higher in the second year than the first (95% CI 1.2, 2.1). The standardised incidence of sero-reversion from positive to negative was 109.5/1000 person-years (95% CI 91.0, 128.0), with lower rates in older age groups and in the second year (Figure S2).

A four-fold or greater IFA titre increase was observed in 50 of 636 initially sero-positive sample pairs tested (incidence rate 81.1/1000 person-years, 95%CI 61.3, 107.5).

### Clinical cohort

**Figure 3:**
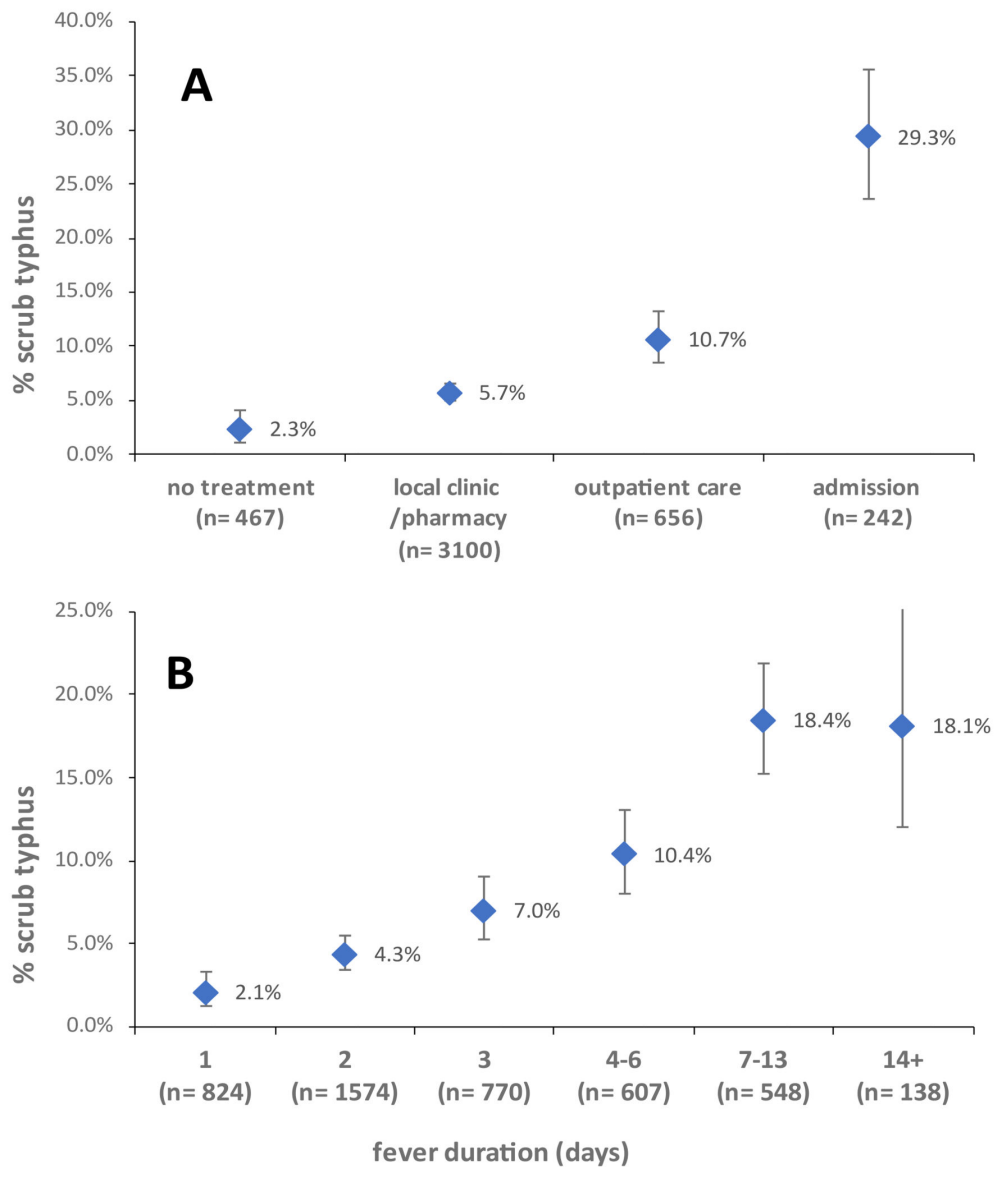
Proportion of scrub typhus cases among fever cases by treatment level (A) and total fever duration (B).

32,279 individuals (Figure S1) from 7619 households were observed over 54,591 person-years (Figure 1) during 13 rounds of follow-up. We observed 6175 fever episodes, with a blood sample taken in 4474 (72%). Of these, 328 episodes (7.3%), occurring in 316 individuals, met the clinical case definition for scrub typhus, and of these 118 (36.0%, Table S1) met the definition of a confirmed case. An eschar was found in 18.6% (61/328) of cases. The proportion of fever cases diagnosed as scrub typhus increased with fever duration and higher care level (Figure 3), with scrub typhus accounting for 29.3% of all fever hospitalizations. The incidence of scrub typhus based on the clinical case definition was 6.0 per 1000 person-years (95% CI 4.8, 7.5). Adjustment for sample unavailability and IgM sero-prevalence in asymptotic controls (1.3%) suggested a rate of 6.6/1000 person-years (Supplement). Case numbers showed a seasonality (Figure S3), and higher rates in the second year (RR 1.4, 95% CI 1.1, 1.8). Seventy-one cases were hospitalized (incidence rate 1.3/1000 person-years, 95%CI 1.0, 1.7). Twenty-nine cases (8.8% of clinical cases, Table) were severe (incidence rate 0.5 per 1000 person-years, 95%CI 0.4, 0.8). Compared to males, females had a higher age-adjusted rate of clinically apparent (RR 1.6, 95%CI 1.3, 2.0), but not of severe infection (RR 1.0, 95%CI 0.5, 2.1). The rate of clinical and severe infection increased with age (Figure 2). Among clinical cases, diabetes was associated with a 2.4-fold increase in the risk of severe infection (Table S3).

Baseline IgG sero-status from a sample taken at the beginning of a year was available in 5891 participants of the sero-cohort and 99 subsequent clinical infections occurred. In these, baseline IgG positivity did not protect against subsequent clinical illness (RR 1.4, 95%CI 0.9, 2.1). Of the 99 cases, 5 were severe, all of whom were sero-negative at baseline (Fisher’s exact p= 0.02).

A verbal autopsy was done in 642 of 645 deaths (Table S4). Five deaths were assessed as due to scrub typhus confirmed by PCR (n= 3) or presence of an eschar (n= 2, Table 1), an incidence rate of 0.09/1000 person-years, 95% CI 0.04, 0.2), and a case fatality of 1.5% (5/328). Thirty-seven fever deaths were assessed as due to SARS-CoV-2. Thirty-three fever deaths were unexplained, mostly coinciding with SARS-CoV-2 waves in India (Figure S4).

Restricting the analysis to participants of the sero-cohort, the standardized incidence of clinical infection was 12.4/1000 person-years (95%CI 8.9, 14.5). The incidence rates of scrub typhus hospitalizations and severe infection were 1.3/1000 person-years (95% CI 0.7, 2.4) and 0.8/1000 person-years (95% CI 0.4, 1.9), respectively.

## Discussion

This cohort study conducted in a highly endemic setting yielded estimates of scrub typhus incidence by severity, allowing us to construct a severity pyramid of this neglected infection. Most infections caused no or minimal symptoms. Among those with symptomatic illness, hospitalisations and severe illness were common. Given that the study was conducted under the unusual circumstances of a pandemic, with SARS-CoV-2 accounting for almost 50% of fever admissions, scrub typhus is likely to be a common cause of fever admission outside pandemic situations in this setting, consistent with earlier hospital-based studies.^5,9–12^ The data on test-positivity by care level and fever duration could inform empirical treatment strategies.

The study also provides insights into the immunity of the infection. Sero-prevalence and sero-incidence across all levels of severity increased with age, possibly because traditional lifestyles involving higher-risk behaviours such as agricultural activities^8,21^ may be more common in those who are older. The increase in IgG sero-prevalence with age is likely due in part to the higher rate of seroconversion and lower rate of re-version in older ages (Figure S2), which we think may be due to repeated infection.^25^

High baseline IgG antibody levels did not protect from clinical infection, in fact, seemed to predispose to it, presumably because higher antibody levels may reflect frequent exposure to the vector. However, despite small numbers, there is a suggestion that baseline IgG sero-positivity is associated with less severe illness. None of the 5 severe cases with available baseline IgG sero-status were IgG sero-positive for scrub typhus prior to the infection. Frequent reversion to sero-negativity after infection means we cannot exclude prior infection in these 5 individuals. On the whole, however, our findings do not support the previous suggestion, by our group, of antibody-dependent enhancement in scrub typhus.^31^ Why older age groups are at the highest risk of severe infection despite higher sero-prevalence merits further study. Immuno-senescence and comorbidities, in particular diabetes^32,33^ may play a role.

In the study area, males and females account for similar numbers of admission for severe scrub typhus,^31^ a finding consistent with the present study where males and females were at an equal risk of severe illness. The higher overall risk of infection in females could imply a higher risk of developing severe illness in males, given infection. This pattern is already evident in other infections, most notably SARS-CoV-2.^34^ As highlighted by the miscarriages in two women with otherwise non-severe scrub typhus, pregnant women may constitute a further vulnerable group.^35,36^ The case fatality of 1.5% is lower compared to hospital-based studies,^12^ presumably due to the inclusion of milder cases in the community.

Under-reporting of febrile illness is perhaps the most important limitation of this study. Given strict SARS-CoV-2 quarantine measures implemented in the study area, this was to be expected. Due to logistical challenges during the pandemic, we were unable to maintain planned visits every 4 weeks. On the other hand, long visit intervals may have reduced changes in treatment-seeking behaviour among study participants. To explore under-reporting, we conducted an unplanned subgroup analysis of clinical incidence in the sero-cohort, probably representing a largely cooperative subgroup of the study population that was older and more often female. This analysis noted rates of clinical infection twice as high as in the main cohort (rates of hospitalisation and severe infection were similar). Additional under-reporting of fevers in the sero-cohort sub-population affecting these figures can, however, not be excluded. By contrast, loss to follow-up was rare and unlikely to affect results.

This study was conducted in a highly endemic setting, representing a typical rural Indian population at risk of scrub typhus.^21^ Clinical characteristics, risk factors and immunology may differ by level of endemicity and strain diversity. Case ascertainment was mainly based on ELISA, which has shown high sensitivity and specificity in this setting.^37^ Probably due to frequent asymptomatic infection, 1.3% of asymptomatic controls were IgM positive. Adjusting for this lowered the incidence, but this was offset by accounting for fever cases not tested. However, this method does not fully account for the possibility of cross-reactivity with other pathogens. We used IFA-based titration to confirm infection in those IgG ELISA positive at two time points. This approach is limited by the difficulty of choosing the fold-increase to define seroconversion.^38^ Seroconversion based on IgG ELISA is easier to define but is subject to the choice of locally applicable cut-off points,^37,39^ and can be problematic if two optical density values differ only by a small amount, straddling the cut-off point. However, a sensitivity analysis using a minimal difference of 0.5 to define seroconversion reduced the incidence by only 11%, from 81.2/1000 to 71.9/1000.

To conclude, this study estimates parameters to help quantify the burden of scrub typhus infection across endemic regions in Asia where large populations are at risk. Such estimates will be needed to calculate the cost-effectiveness of vaccines, the development of which has not yet advanced very far.^40^ The high proportion of hospitalized febrile illnesses attributable to scrub typhus demonstrated in this study underscores the need for more research^14,41^ and medical education as a common cause of undifferentiated febrile illness.

## Supplementary Material

Supplement

## Figures and Tables

**Figure 4:**
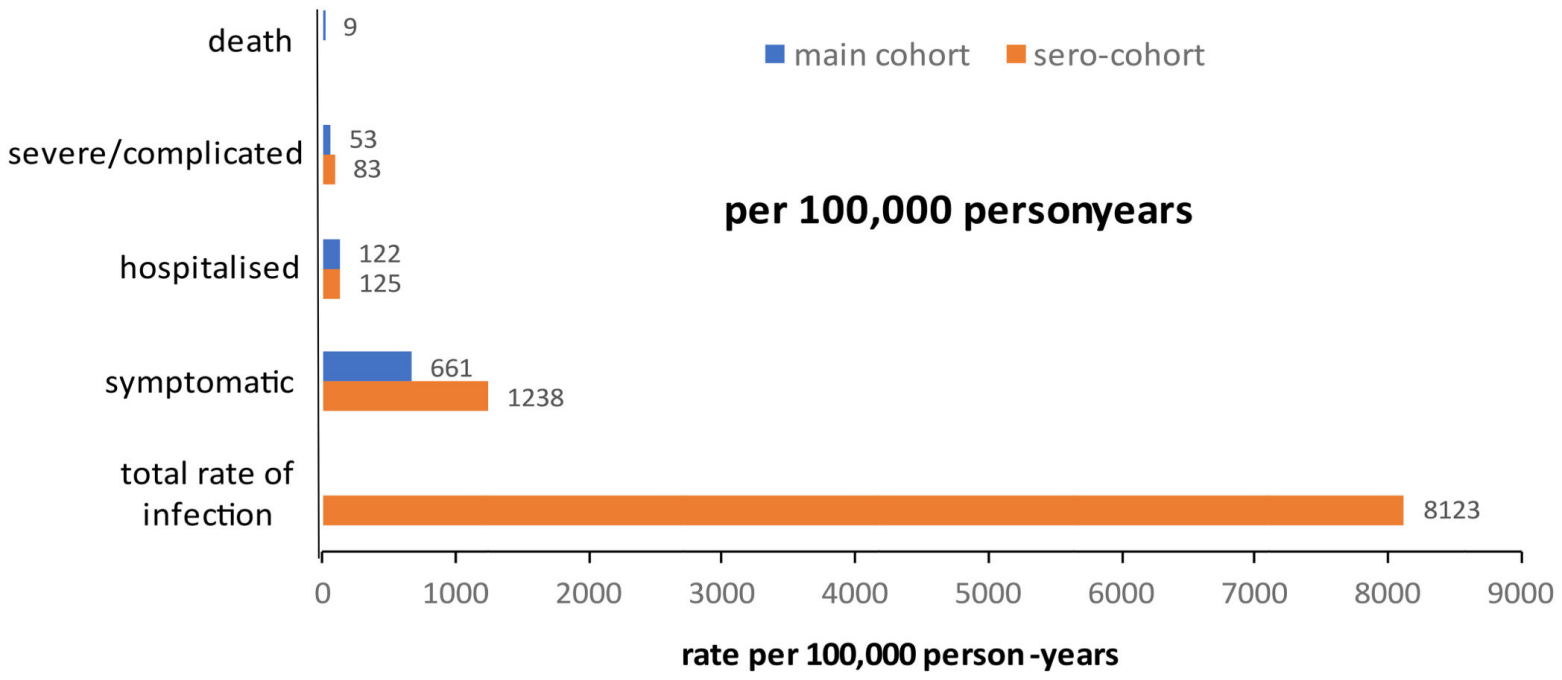
Summary of the rates of scrub typhus infection per 100,000 person years by level of severity estimated from the main cohort (blue) and sero-cohort (orange). The estimate for seroconversion was used to represent total rate of infection.

**Table:** Clinical characteristics of severe cases

	N (%)	% confirmed case (n/N)	Case details
**Clinical features**			
ARDS	19 (66%)	79% (15/19)	
CNS	4 (14%)	75% (3/4)	Encephalitis (2x), Meningitis with cranial nerve palsy (2x)
Shock	11 (38%)	55% (6/11)	
Acute kidney injury	6 (21%)	50% (3/6)	Dialysis done in 1 case
Myocarditis	2 (7%)	100% (2/2)	
Miscarriage	2 (7%)	50% (1/2)	Miscarriage at 8 weeks during fever (1x), Miscarriage at 7 weeks one week after fever (1x)
Death	5 (17%)	100% (5/5)	Multi-organ failure (4x – 29y female, 42y male, 50y female, 70y male) Sudden death (1x – 80y female)
**Respiratory** **support**			
High flow O2 via mask/nasal prong	13 (45%)	50% (4/4)	
Non-invasive ventilation	3 (10%)	100% (3/3)	
Invasive ventilation	5 (17%)	80% (4/5)	
