# Supplementary material for: Incidence of Scrub Typhus in Rural South India
Source: N Engl J Med. Author manuscript; Available in PMC 2025 Mar 19. (PMC7617504; doi:10.1056/NEJMoa2408645)
Supplement: Supplement [file EMS202091-supplement-Supplement.pdf]

Supplementary Material to "Incidence of Scrub Typhus in Rural South India: Cohort Study" by Devamani et al.

Table of Contents

1. Author contributions 2
2. Laboratory procedures 2
2.1. Testing for clinical scrub typhus infection using IgM ELISA 2
2.2. Testing for clinical scrub typhus infection using real-time PCR 2
2.3. Confirmation of clinical scrub typhus infection by IgG ELISA and IFA 3
2.4. Testing for serological scrub typhus infection using IgG ELISA (sero-cohort) 3
2.5. Testing for serological scrub typhus infection using IFA (sero-cohort) 3
3. Statistical analysis 4
3.1. Adjusting incidence rate for fever cases without IgM ELISA result 4
3.2. Adjusting incidence rate for IgM ELISA sero-prevalence in asymptomatic controls 5
3.3. Calculation of standardized incidence rates in sero-cohort 5
4. Description of organ involvement used to define severe infection 7
5. Reversion from sero-positive to sero-negative in sero-cohort 8
6. Seasonality of scrub typhus 8
7. Comorbidities and severe infection 9
8. Causes of death 10
9. Comparison between the sero-cohort and main cohort 11
10. Characteristics of study participants lost to follow-up 11
11. Representativeness of Study Participants 12
12. References 13

1. Author contributions

Conception or design of the study: Carol Devamani, Neal Alexander, Daniel Chandramohan, Mary Cameron, Punam Mangtani, Wolf-Peter Schmidt

Acquisition of the data: Carol Devamani, Neal Alexander, Kundavaram PP Abhilash, John Stenos, Stuart Blacksell, Huong Thi Thu Vu, Winsley Rose, Wolf-Peter Schmidt

Analysis of the data: Carol Devamani, Neal Alexander, Huong Thi Thu Vu, Wolf-Peter Schmidt

Vouches for the data and the analysis: All authors

Wrote the first draft of the manuscript: Carol Devamani and Wolf-Peter Schmidt

Wrote the paper (reviewing and editing): All authors

Decision to publish the paper: All authors

2. Laboratory procedures

2.1. Testing for clinical scrub typhus infection using IgM ELISA

At each household visit, a venous blood sample was taken from acute or convalescent fever cases occurring since the last visit. After collection, blood samples were brought to the study center on the same day. Serum was separated from blood cells, divided into up to 3 aliquots and stored at -70°C until testing. We used enzyme-linked immunosorbent assays (ELISA) to detect IgM antibodies to *Orientia tsutsugamushi* (Scrub Typhus Detect, InBios International, Inc., Seattle, WA, USA) following the manufacturer's instructions. This ELISA uses Karp, Kato, Gilliam and TA716 recombinant proteins of the 56-kD outer membrane protein as antigen captures. This assay has been shown to have a 92% sensitivity and 94% specificity in a study done at the location of the present study.[1] All assays were performed using an automated ELISA analyser (Euroimmun Analyzer1, Euroimmun, Lübeck, Germany). Quality control measures included monthly calibration of the ELISA workstation. With every run, we included an internal control and a split sample in addition to the kit controls. We applied an optical density (OD) cut-off of ≥1.0 for IgM to suggest acute scrub typhus infection, which is used for routine diagnostics at the study institution. We have previously shown that in the vast majority of cases, *O. tsutsugamushi* IgM remains above this cut-off for 6 to 12 weeks following infection.[2]

2.2. Testing for clinical scrub typhus infection using real-time PCR

In all ongoing fever cases, we took an additional EDTA blood sample. After centrifuging, we used the buffy coat for DNA extraction (HiYield Genomic DNA Mini Kit, RBC, Taiwan). The qPCR assay targeted the 47-kDa protein gene according to Jiang et al.[3] The final primer concentration (forward: OtsuFP630 - 5'-AAC TGA TTT TAT TCA AAC TAA TGC TGC T-3'; reverse: OtsuRP747 - 5'-TAT GCC TGA GTA AGA TAC RTG AAT RGA ATT-3') was 0.1 µM. The final TaqMan probe concentration (OtsuPR665 – 5'-6FAM-TGG GTA GCT TTG GTG GAC CGA

TGT TTA ATC T-BHQ1-3') was 0.2 µM. The final reaction volume of 25 µl contained 15 µl of Platinum Quantitative PCR SuperMix-UDG (Invitrogen) and 1 µl DNA template. The PCR cycling conditions were: hold at 50°C for 2 min, hold at 95°C for 2 min, followed by 45 cycles of 95 °C for 15 seconds (denaturation) and 60 °C for 30 sec (Combined annealing/extension). A ct value of less than 39 was regarded as positive. If the ct value was between 39 and 41, the assay was repeated and treated as positive if the second ct value was below 40.

2.3. Confirmation of clinical scrub typhus infection by IgG ELISA and IFA

In scrub typhus cases with an acute and a convalescent sample we used IgG ELISA (Scrub Typhus Detect, InBios International, Inc., Seattle, WA, USA) as above to identify IgG seroconversion from an OD of <1.0 to ≥1.0 (Table S1). In scrub typhus cases that were IgG ELISA positive in the acute and the convalescent sample, we performed indirect immuno-fluorescence assays (IFA) to confirm infection, based on a 4-fold titre or greater increase in the IgG titres from the acute to the convalescent sample to a titre of at least 1:128 (see 1.5. for details).

2.4. Testing for serological scrub typhus infection using IgG ELISA (sero-cohort)

We tested for serological infection between two time points in the serological cohort irrespective of reported fever symptoms. Similar to 1.3. all samples from the sero-cohort were tested for IgG using InBios ELISA to identify IgG seroconversion from an OD of <1.0 to ≥1.0.

2.5. Testing for serological scrub typhus infection using IFA (sero-cohort)

We used indirect immuno-fluorescence assays (IFA) to titrate dual IgG ELISA positive sample pairs as the most commonly used method to quantify antibody levels in scrub typhus.[4] IFA slides were acquired from the Australian Rickettsia Reference Lab (Geelong, Victoria, Australia) and contained the Karp, Kato and Gilliam strains of *O. tsutsugamushi*. Sera were diluted to 1:64 using 2% casein PBS buffer, and then titrated pairwise or in triplicate (depending on whether samples ELISA were IgG positive at only two or all three consecutive timepoints) up to a titre of 1:8192 before adding the samples to the IFA slides. Anti-human IgG (H+L) antibody FITC-labelled conjugate (Sera Care) was added to mark antigen-antibody complexes. The slides were subsequently washed, dried and observed microscopically (400x magnification) by two observers. Differences between the ratings between the two observers were resolved through discussion. Infection was defined as a 4-fold or greater titre increase in the IgG titres from one year to the following year to a titre of at least 1:128.

Applying a 4-fold or greater titre increase to determine infection is a commonly used criterion, mainly based on the technical difficulties in most titration assays of identifying a 2-fold titre increase with sufficient accuracy.[5,6] IFA is further limited by considerable inter-observer variability.[7] Due to logistical challenges in scaling up the time-consuming procedures to prepare and read IFA slides, we restricted IFA in the sero-cohort to a random sample of 25% of dual IgG ELISA positive sample-pairs.

Table S1. Case confirmation by method (328 clinical cases)

Method	N (%)
Total test results	328 (100%)
IgM ELISA+	313 (95.4%)
IgG ELISA seroconversion	49 (14.9%)
IFA IgG ≥4 fold titer increase	30 (9.2%)
Eschar	61 (18.6%)
PCR+	31 (9.5%)
Total confirmed cases	118 (36.0%)
IgM ELISA positive + eschar	42 (12.8%)
IgM ELISA positive + PCR positive +/- eschar	8 (2.4%)
IgM ELISA positive + IgG sero-conversion/IFA titer increase +/- eschar	45 (13.7%)
IgM ELISA positive + IgG sero-conversion/IFA titer increase + PCR positive +/- eschar	9 (2.7%)
PCR positive	14 (4.3%)
Total probable cases	210 (64.0%)
IgM ELISA positive	209 (63.7%)
Eschar	1 (0.3%)

*ELISA – enzyme linked immunosorbent assay; IFA - indirect immuno-fluorescence assay; PCR – polymerase chain reaction

3. Statistical analysis

3.1. Adjusting incidence rate for fever cases without IgM ELISA result

No test results were available in 1701 of 6175 fever cases (28%). To predict the aggregate number of expected scrub typhus IgM ELISA positive cases in this group, we used a logistic regression model to predict the probability of a positive IgM ELISA based on variables found to be highly associated with IgM positivity: age, sex, number of fever days, care level (Figure 3) and calendar time (modeled as cubic spline with 6 knots chosen following Harrell[8]). In other words, we used logistic regression in those with data on both the outcome (IgM status) and the covariates, to estimate the probability of the former as a function of the latter. We extrapolated this model to estimate the probability of the outcome for those who had data only on the covariates. We then summed those probabilities for these 1701 individuals to arrive at the predicted aggregate number of IgM positive fever cases. This resulted in 107 predicted IgM-positive fever cases (a proportion of 107/1701= 6.3%). When added to the 328 scrub typhus cases among those that were tested, the total number of cases was 435.

3.2. Adjusting incidence rate for IgM ELISA sero-prevalence in asymptomatic controls

Earlier studies have suggested that a small percentage of people without a recent history of a febrile illness in the study area may be positive for scrub typhus IgM,[9] for example following asymptomatic infection or due to persisting IgM antibodies from scrub typhus episodes having occurred in the more distant past. To estimate the sero-prevalence of scrub typhus IgM positivity in study participants without recent fever, we obtained concurrent blood samples from a consenting study participant residing in the same or neighboring household of a fever case identified during follow up. Those who did not have an episode of febrile illness in the last 6 months were eligible for enrolment as control. We collected a total of 947 control samples, of which 12 were positive for scrub typhus IgM (1.3%). By contrast, the predicted IgM sero-positivity in fever cases was 435/6175 = 7.0%.

Similar to studies for example on the etiology of diarrhoeal diseases[10] we used population attributable fractions (PAF) to adjust the proportion of fever cases diagnosed as scrub typhus (7.0%) for the IgM sero-positivity in controls (1.2%), using the following standard formula for case-control studies:[11]

$PAF = 1 - (b/n_1)/(d/n_0)$

Where:

	IgM positive	IgM negative	Total
**Fever cases**	a = 435	b = 5740	n1 = 6175
**Controls**	c = 12	d = 935	n0 = 947

Based on this formula, the population attributable fraction, in this case, the proportion of fever cases attributable to scrub typhus is 5.8%, implying that only 361 of 435 predicted scrub typhus cases were due to scrub typhus. The corresponding incidence rate is 6.6/1000 person-years.

3.3. Calculation of standardized incidence rates in sero-cohort

The demographic characteristics of the sero-cohort (a subset of the main cohort) differed substantially from the main cohort (Figure S1), with females over-represented and younger ages under-represented. This was probably due to differences in the willingness to provide regular blood samples across different ages and by sex and/or in the likelihood of being present at the location at the time of the visit. Further, the size of the sero-cohort population decreased in the second year while the population size of the main cohort remained approximately constant. Therefore, the estimates for the incidence rate of serological infection needed to be standardized to the age/sex characteristics of the main cohort to allow comparability. For the standardization we constructed 4 age groups (0-19, 20-39, 40-59 and 60+) for each sex, and each year.

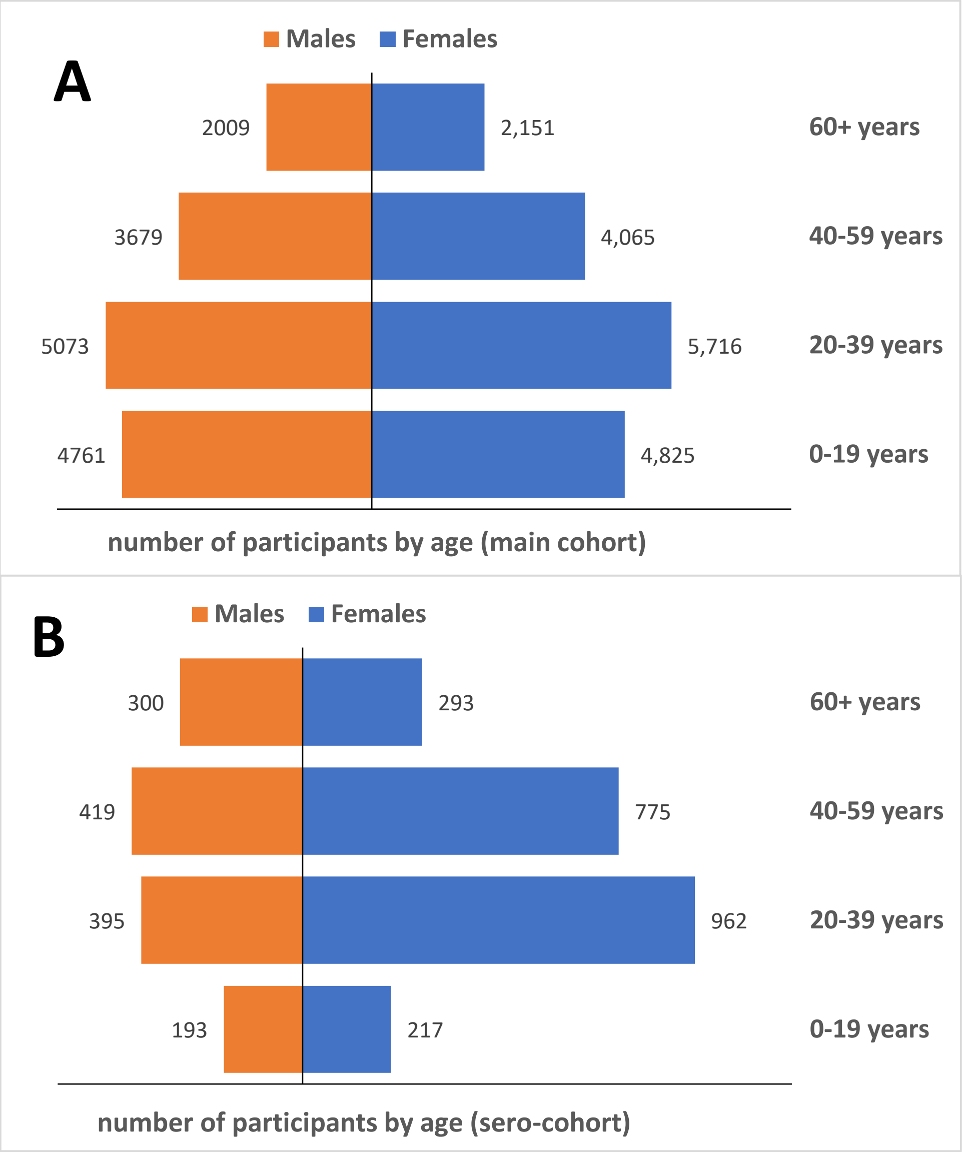

Figure S1. Age structure of study participants by sex. A – main cohort, B – sero-cohort

The estimation of the incidence rate for IgG ELISA seroconversion in the sero-cohort was restricted to individuals sero-negative at the beginning of a year in the study between two consecutive blood samples, as only these were at risk of seroconversion. Therefore, this rate was standardized using the expected number of IgG sero-positive study participants of the main cohort, estimated based on the age-specific IgG sero-prevalence found in the 5602 study participants with a baseline blood sample (Figure 1). Similarly, the rate of re-conversion (from sero-positive to sero-negative) only applied to sero-positive individuals at the beginning of a period under study. The rate was, therefore, standardized using the estimated number of IgG sero-positive individuals in the main cohort.

Direct standardization and calculation of confidence intervals of standardized rates were performed using the STATA command *dstdize*.

4. Description of organ involvement used to define severe infection

Table S2: Definitions of organ involvement

Organ system	Definition
**Lung involvement**	Lung involvement –oxygen saturation below 92% and tachypnea at any time during admission. Tachypnea was defined as >20 breaths/minute in adults; children: >30/min aged 2-5 years, >25/min 5-12 years.
**Shock**	Adults: documented hypotension (systolic <90mmHg) at presentation or during treatment not responding to a single fluid bolus, or any documented use of inotropes. Children: documented hypotension <80mmHg (2-5 years), <90mmHg (5-12 years), or any documented use of inotropes; or capillary refill time >2s with tachycardia >150/min (2-5 years), >130/min (5-12 years).
**Myocarditis**	New onset heart failure confirmed by echocardiography with an elevated troponin T in a patient with no known heart condition.
**Vascular**	Large vessel occlusion, eg. peripheral gangrene or organ infarct.
**Kidney injury**	Any creatinine of 3.0 mg/dl or higher in the absence of a known, pre-existing chronic kidney disease.
**Central nervous system (CNS)**	Any focal neurological deficit, or any elevated white blood cell counts in a cerebrospinal fluid sample, or any focal or generalised seizure in an adult, or any focal or generalised seizure in a child not diagnosed as simple febrile seizure. Simple febrile seizure in children less than 6 years of age was assumed if there was no more than one generalised seizure lasting less than 15 minutes.
**Severe bleeding manifestation**	Purpura fulminans, gastro-intestinal or urinary tract haemorrhage.
**Death**	Any natural death during the hospital stay.

5. Reversion from sero-positive to sero-negative in sero-cohort

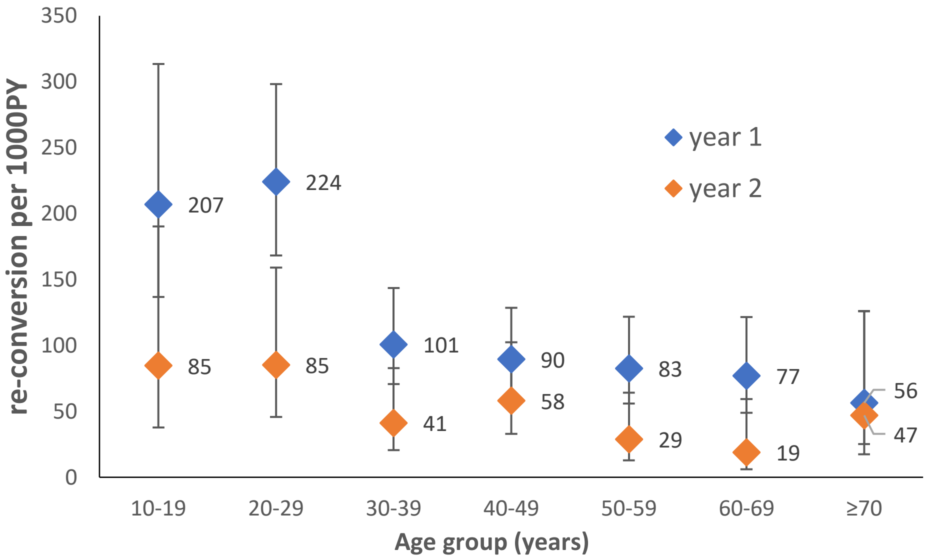

Figure S2. Incidence rates of reversion from sero-positivity to sero-negativity in sero-cohort by age and year. The Figure shows a marked decrease in reversion with age, as opposed to the marked increase in sero-incidence with age. Reversion was lower in the second year than the first (RR 0.4, 95%CI 0.3, 0.6)

6. Seasonality of scrub typhus

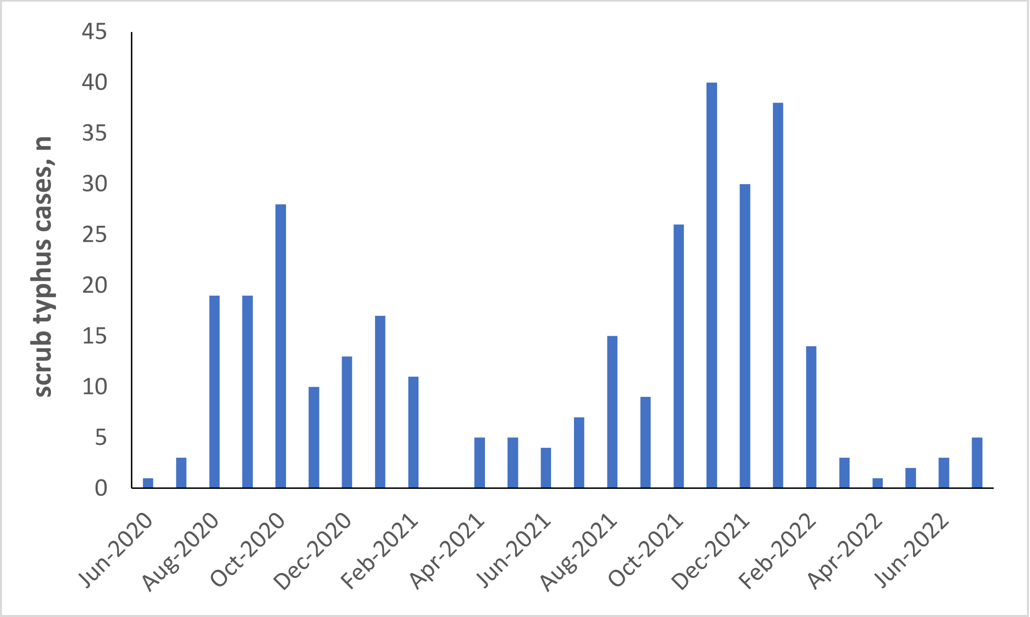

Figure S3. Seasonality of scrub typhus. The Figure demonstrates the typical seasonality of scrub typhus in the study area as described previously,[12,13] and confirms the potential for marked variations of the incidence between years.

7. Comorbidities and severe infection

Table S3. Association between comorbidities and severe infection in clinical cases

	**% severe infection (n/N)**	**Crude RR**	**95%CI**	**Adjusted RR***	**95% CI**
Diabetes					
No	7.4% (20/270)	1.0 (ref)		1.0 (ref)	
Yes	19.2% (5/26)	2.6	1.1, 6.3	2.4	0.9, 6.3
Hypertension					
No	7.8% (20/258)	1.0 (ref)		1.0 (ref)	
Yes	13.6% (5/38)	1.7	0.7, 4.3	1.6	0.6, 4.3

*adjusted for age and sex

8. Causes of death

Table S4. Causes of death

Cause	n/N (%)
Total	645
Deaths with verbal autopsy	642 (100%)
Road traffic accidents	27 (4.2%)
Other accidents	26 (4.0%)
Suicide	20 (3.1%)
Medical cause – non febrile	484 (75.4%)
Medical cause – febrile	87 (13.6%)
Local fever source	9 (1.4%)
Undifferentiated fever	76 (11.8%)
SARS-CoV-2	37 (5.8%)
Scrub typhus	5 (0.8%)
Spotted Fever rickettsiosis	1 (0.2%
Cause not identified	33 (5.1%)

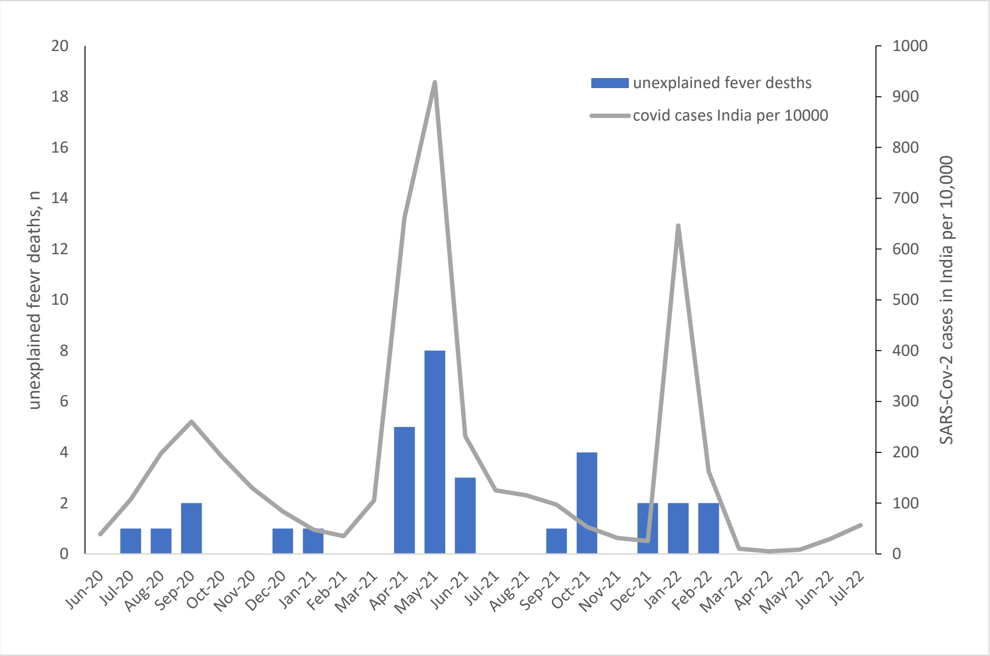

Figure S4. Unexplained fever deaths (n= 33) and SARS-CoV-2 in India (from[14]).

9. Comparison between the sero-cohort and main cohort

Table S5. Comparison between participants of the sero-cohort and participants not part of the sero-cohort

	participants of the sero-cohort	participants not part of the sero-cohort	P-value
**N (%)**	3554 (11.0%)	28725 (89.0%)	-
**Females, n (%)**	2248 (62.2%)	14509 (50.5%)	<0.001
**Age, mean (SD)**	40.6 (16.6%)	31.8 (20.6)	<0.001
**Scrub typhus cases, n**	80	248	-
**Total person-years**	6405	48181	<0.001
**Person-years per participant**	1.80	1.67	<0.001
**Incidence rate**	12.4 per 1000 PY	5.1 per 1000 PY	<0.001
**Duration of scrub typhus fever, mean (SD)**	6.0 (5.2)	5.9 (4.4)	0.783
**Cases hospitalized, n/%**	9 (9.9%)	62 (25.0%)	0.009
**Cases severe infection, n/%**	6 (7.5%)	23 (9.3%)	0.627

10. Characteristics of study participants lost to follow-up

Table S6 shows the comparison of study participants leaving the study early for any reason other than death with participants remaining in the study or dying during the study. Participants leaving the study early were more often female and of younger age, probably reflecting women leaving their household due to marriage and women after childbirth who in this setting often go back to their own family's home for several months.

Table S6. Characteristics of study participants lost to follow-up

	Lost to follow-up	Remained in study	P
**N**	1692 (5.2%)	30587 (94.8%)	
**Females, n (%)**	974 (57.6%)	15783 (51.6%)	<0.001
**Age, mean (SD)**	27.4 (17.8)	33.1 (20.5)	<0.001
**Clinical incidence rate**	6.5 per 1000 PY	6.0 per 1000 PY	0.802

11. Representativeness of Study Participants

Table S7. Representativeness of Study Participants

**Category**	**Details**
Disease under investigation	Scrub typhus, a mite-borne infection caused by *Orientia tsutsugamushi*, an intracellular bacterium
Sex and gender	Scrub typhus affects both males and females, but women may be at a higher risk of infection during household and agricultural activities. Males may be more prone to severe infection, but females contracting scrub typhus during pregnancy may experience poor pregnancy outcomes.[15-17]
Age	Scrub typhus disproportionally affects elderly people, especially in its severe form, possibly due to immune-senescence and comorbidities.
Race or ethnic group	Marginalized indigenous people such as hill tribes in Thailand[18] and Malaysia[19] may have a higher burden of scrub typhus. In India, rural populations in lowland agricultural regions seem to have a high risk of scrub typhus independent of ethnic or caste background.[20]
Geography	Scrub typhus is endemic to large parts of East Asia, South East Asia, South Asia, as well as Northern Australia and the Arabic Peninsula. Scrub typhus has recently been discovered in Chile. Trombiculid mite larvae (chiggers) the vectors of scrub typhus, occur in a wide range of habitats, but appear to be especially common in disturbed habitats such as forest edges and agricultural fields. The have also been found around human settlements and even city parks.
Overall representativeness of this trial	The participants in the overall trial had a near equal ratio of males to females and were of all ages, but females and older age groups, which are more prone to infection, predominated in the sero-cohort.<br>The study area represents low land agricultural regions in South India, but possibly elsewhere, that have the highest burden of scrub typhus.[20] However, scrub typhus occurs in a wide range of geographies including urbanized regions not represented in this study.<br>The study villages were chosen based on a known high endemicity for scrub typhus. The level of acquired immunity is likely to differ by level of endemicity, possibly influencing the epidemiology of the infection such as the proportion of infections leading to severe infection.

12. References

1. Kannan K, John R, Kundu D, et al. Performance of molecular and serologic tests for the diagnosis of scrub typhus. PLoS Negl Trop Dis 2020;14(11):e0008747. DOI: 10.1371/journal.pntd.0008747.
2. Schmidt WP, Devamani CS, Rose W, Alexander N, Prakash JAJ. Antibody response following scrub typhus infection: clinical cohort study. Trop Med Int Health 2019;24(12):1455-1464. DOI: 10.1111/tmi.13322.
3. Jiang J, Chan TC, Temenak JJ, Dasch GA, Ching WM, Richards AL. Development of a quantitative real-time polymerase chain reaction assay specific for Orientia tsutsugamushi. Am J Trop Med Hyg 2004;70(4):351-6. (https://www.ncbi.nlm.nih.gov/pubmed/15100446).
4. Blacksell SD, Bryant NJ, Paris DH, Doust JA, Sakoda Y, Day NP. Scrub typhus serologic testing with the indirect immunofluorescence method as a diagnostic gold standard: a lack of consensus leads to a lot of confusion. Clin Infect Dis 2007;44(3):391-401. DOI: 10.1086/510585.
5. Alexander N, Schmidt WP. Agreement and error of titration assays. J Immunol Methods 2022;502:113210. DOI: 10.1016/j.jim.2021.113210.
6. Cauchemez S, Horby P, Fox A, et al. Influenza infection rates, measurement errors and the interpretation of paired serology. PLoS Pathog 2012;8(12):e1003061. (In eng). DOI: 10.1371/journal.ppat.1003061PPATHOGENS-D-12-01960 [pii].
7. Phetsouvanh R, Thojaikong T, Phoumin P, et al. Inter- and intra-operator variability in the reading of indirect immunofluorescence assays for the serological diagnosis of scrub typhus and murine typhus. Am J Trop Med Hyg 2013;88(5):932-936. DOI: 10.4269/ajtmh.12-0325.
8. Harrell FEj. Regression Modeling Strategies: With Applications to Linear Models, Logistic Regression, and Survival Analysis. New York: Springer, 2001.
9. Devamani CS, Prakash JAJ, Alexander N, Suzuki M, Schmidt WP. Hospitalisations and outpatient visits for undifferentiated fever attributable to scrub typhus in rural South India: Retrospective cohort and nested case-control study. PLoS Negl Trop Dis 2019;13(2):e0007160. DOI: 10.1371/journal.pntd.0007160.
10. Kotloff KL, Nataro JP, Blackwelder WC, et al. Burden and aetiology of diarrhoeal disease in infants and young children in developing countries (the Global Enteric Multicenter Study, GEMS): a prospective, case-control study. Lancet 2013;382(9888):209-22. DOI: 10.1016/S0140-6736(13)60844-2.
11. Schlesselman JJ. Case-control studies: Design, conduct, analysis. . Oxford: Oxford University Press, 1982.
12. D'Cruz S, Sreedevi K, Lynette C, Gunasekaran K, Prakash JAJ. Climate influences scrub typhus occurrence in Vellore, Tamil Nadu, India: analysis of a 15-year dataset. Sci Rep 2024;14(1):1532. DOI: 10.1038/s41598-023-49333-5.
13. Varghese GM, Raj D, Francis MR, Sarkar R, Trowbridge P, Muliyil J. Epidemiology & risk factors of scrub typhus in south India. Indian J Med Res 2016;144(1):76-81. (In eng). DOI: 10.4103/0971-5916.193292IndianJMedRes_2016_144_1_76_193292 [pii].
14. Our world in Data. (https://ourworldindata.org/coronavirus/country/india).
15. McGready R, Prakash JA, Benjamin SJ, et al. Pregnancy outcome in relation to treatment of murine typhus and scrub typhus infection: a fever cohort and a case

series analysis. PLoS Negl Trop Dis 2014;8(11):e3327. (In eng). DOI: 10.1371/journal.pntd.0003327PNTD-D-14-00535 [pii].

16. Rajan SJ, Sathyendra S, Mathuram AJ. Scrub typhus in pregnancy: Maternal and fetal outcomes. Obstet Med 2016;9(4):164-166. DOI: 10.1177/1753495X16638952.
17. Yadav B, Soni R, Biswal M, Suri V, Rohilla M. Clinical profile and outcomes of Scrub typhus in pregnant women presenting to a tertiary care hospital of North India. J Obstet Gynaecol 2023;43(1):2141617. DOI: 10.1080/01443615.2022.2141617.
18. Tasak N, Apidechkul T, Law ACK, et al. Prevalence of and factors associated with scrub typhus exposure among the hill tribe population living in high incidence areas in Thailand: a cross-sectional study. BMC Public Health 2023;23(1):2394. DOI: 10.1186/s12889-023-17313-z.
19. Brown GW, Robinson DM, Huxsoll DL. Serological evidence for a high incidence of transmission of Rickettsia tsutsugamushi in two Orang Asli settlements in Peninsular Malaysia. Am J Trop Med Hyg 1978;27(1 Pt 1):121-3. (In eng) (http://www.ncbi.nlm.nih.gov/pubmed/415625).
20. Devamani CS, Schmidt WP, Ariyoshi K, Anitha A, Kalaimani S, Prakash JAJ. Risk Factors for Scrub Typhus, Murine Typhus, and Spotted Fever Seropositivity in Urban Areas, Rural Plains, and Peri-Forest Hill Villages in South India: A Cross-Sectional Study. Am J Trop Med Hyg 2020. DOI: 10.4269/ajtmh.19-0642.
